# Influence of Hydroxytyrosol Acetate Enrichment of an Oil Rich in Omega-6 Groups on the Evolution of Its Oxidation and Oxylipin Formation When Subjected to Accelerated Storage. A Global Study by Proton Nuclear Magnetic Resonance

**DOI:** 10.3390/antiox11040722

**Published:** 2022-04-06

**Authors:** Sofía del Caño-Ochoa, Ainhoa Ruiz-Aracama, María D. Guillén

**Affiliations:** Food Technology, Faculty of Pharmacy, Lascaray Research Centre, University of the Basque Country (UPV-EHU), Paseo de la Universidad n 7, 01006 Vitoria-Gasteiz, Spain; sofia.delcano@ehu.eus (S.d.C.-O.); ainhoa.ruiz@ehu.eus (A.R.-A.)

**Keywords:** edible oil, polyunsaturated acyl groups, phenolic compound enrichment, accelerated storage, oxidation process, ^1^H NMR spectroscopy, linoleic acyl group degradation, oxidation compounds, antioxidant effect

## Abstract

Sunflower oil samples, both unenriched and enriched with four different concentrations of hydroxytyrosol acetate, were subjected to accelerated storage at 70 °C until a very advanced oxidation stage and the process was monitored by ^1^H NMR spectroscopy. The aim of the study is to know the effect that the presence of this antioxidant has on the oxidation process of sunflower oil under the aforementioned conditions, as well as on the formation and evolution of the concentration of a significant number of oxylipins. The oxidation process was studied globally by monitoring, during storage time, the degradation of both the linoleic acyl group of sunflower oil, which is the main component of sunflower oil, and the added hydroxytyrosol acetate. Simultaneously, the identification of up to twenty-six different types of oxylipins formed in the oxidation process and the monitoring of the evolution of their concentration over the storage time were carried out. In this way, essential information about the effect that hydroxytyrosol acetate provokes on the oxidation of this oil rich in omega-6 polyunsaturated acyl groups, has been obtained. It has also been shown that the enrichment of sunflower oil with this antioxidant under the conditions tested does not prevent the oxidation process but slows it down, affecting the entire oxidation process.

## 1. Introduction

Currently, it is well known that the food industry requires compounds with capacity to delay processes such as lipid oxidation. This process reduces not only the nutritional value and shelf life of foods, but also their safety, due to the generation of toxic compounds [1,2]. As a result there has been great interest in the search for efficient antioxidants. Furthermore, consumers prefer natural antioxidants to synthetic ones, because the latter are perceived as potentially harmful, whereas the former have been attributed beneficial effects on human health. Indeed, food consumption patterns with a high intake of products rich in minor components with potential antioxidant activity are related to a lower incidence of certain pathologies such as cardiovascular disease or cancer [3,4].

In this context, extra virgin olive oil, one of the key products of the Mediterranean diet, is characterized by its resistance against oxidation. This is due to the combination of its high content of oleic acyl groups and its richness in minor components of known antioxidant ability, which are associated with the multiple beneficial health effects of this oil [5,6,7,8]. Among the minority components of this oil, phenolic compounds have the highest antioxidant capacity of all. One of them is hydroxytyrosol acetate, HTy-Ac, detected in this oil for the first time in 1999 [9]. It has no bitter taste unlike other phenolic components of extra virgin olive oil, it is more soluble in lipophilic phases than its hydrolyzed form hydroxytyrosol, and both are among the most abundant phenolic compounds in this oil [10,11].

The biological activity of HTy-Ac has been the subject of numerous studies that have shown that this compound produces multiple beneficial health effects [12]. These include the ability to prevent several diseases, such as lupus erythematosus and acute colitis in mice [13,14], and to regulate the production of joint erosion mediators in human synovial cells [15]. Likewise, this compound has also been reported to be antimicrobial [16,17], to cause platelet antiaggregation [18,19], to be neuroprotective [20], and to have anti-inflammatory [21,22] and antioxidant capabilities. The latter capacity has been considered to be behind many of these health promoting properties. In fact, it is well known that many of the natural products that have antioxidant capacity also have antimicrobial capacity [23].

The antioxidant ability of HTy-Ac has been shown by different assays. Thus, its ability to scavenge some radicals such as DPPH [24,25,26], ABTS [25,27], superoxide anion and hydroxyl, as well as nitric oxide and hydrogen peroxide has been proved [26]. Chelating activity also contributes to inhibiting or delaying lipid oxidation and HTy-Ac has this ability [26]. Likewise, the ferric ion reducing power of HTy-Ac has also been proved by the FRAP assay [25,27] and its antioxidant capacity against peroxyl radical has been tested by the ORAC method [26].

In addition, the antioxidant ability of HTy-Ac has also been demonstrated by the delay, measured by certain parameters that its presence causes in the oxidation of lipid systems enriched with this compound. Thus, this capability has been proved in lipid systems, such as olive oil triglycerides submitted to 60 °C in the dark, controlling the time necessary to reach a fixed Peroxide Value and also determining Anisidine Values [24]. Likewise, this capability of HTy-Ac has also been proved, in the same lipid matrix mentioned above, subjected to 100 °C, 90 °C and 80 °C in a Rancimat apparatus. This measures the so-called oxidation induction period, for the time required for an increase in conductivity caused by volatile secondary or further oxidation compounds, among which is formic acid [27,28,29]. Likewise, the antioxidant ability of HTy-Ac has been evidenced in fish oil submitted to 40 °C, monitoring the formation of Conjugated Dienes by UV spectroscopy [25].

Furthermore, the antioxidant capacity of HTy-Ac has also been demonstrated in ex vivo models. Thus, the HTy-Ac ability to protect proteins and lipids present in a brain homogenate against oxidation caused by peroxyl radicals has been estimated by monitoring the formation of carbonyl groups in the first case and by the TBA test in the second case [29]. Likewise, in the human neuroblastoma cell line SH-SY5Y, HTy-Ac has shown ability to protect the SH group and proteins against oxidation, proving the antioxidant power of this compound [26].

All these assays, with their advantages and limitations, have proved that, under the conditions tested in each case, HTy-Ac has an antioxidant power of a similar order to that of hydroxytyrosol. However, the information extracted from these tests does not go beyond the value of the parameter measured in each case. They do not provide information on the evolution of the lipid oxidation process in the presence of the antioxidant, nor to what extent it favours or hinders the formation of certain oxidation compounds, which are, in short, responsible for biological activity.

In this context, it should be remembered that lipid systems, such as edible oils rich in polyunsaturated acyl groups, which contain natural components of known antioxidant ability, oxidize when kept under oxidative conditions. As a consequence, both fatty acyl groups of the main oil components and minor oil components of known antioxidant capacity degrade simultaneously and, at the same time, the formation of compounds from the degradation of both components occurs [30,31,32]. This means that in the presence of compounds with antioxidant ability, oil oxidation occurs, and for this reason it could be thought that the role of compounds with antioxidant capacity goes beyond the retardation of lipid oxidation, and that it could affect the formation of oxylipins.

Considering all the above, the aim of this study is to clarify whether the enrichment of lipids in HTy-Ac not only increases their oxidative stability by delaying their oxidation but also influences the course of the oxidation process as well as the formation of the different oxidation compounds in a real system. The lipid system chosen is sunflower oil, an oil rich in polyunsaturated omega-6 acyl groups, in which HTy-Ac is absent, which has a high tendency to oxidize. This oil will be enriched with different concentrations of HTy-Ac and will be submitted to accelerated storage conditions at 70 °C. The monitoring of the oxidation process will be carried out by ^1^H NMR spectroscopy and will focus on the degradation of both the main components of the oil and the added HTy-Ac and also on the formation of oxylipins throughout the oxidation process. Regarding lipid oxidation in the presence of antioxidants, it has been accepted that the induction period, or initiation phase, in which oxidation proceeds at a very low rate, continues until the antioxidant has been almost completely consumed, after which the rate of oxidation of the lipid enriched with antioxidant is equal to or even greater than that of the lipid not enriched with antioxidant [33]. However, recent results from other authors referring to the oxidation of pure triglycerides of several edible oils do not agree with the idea postulated above [34] and the results of the study planned here could shed light on this issue in a real lipid system. The interest of this study also lies in the fact that, to the best of our knowledge, it is the first of these characteristics and all the information it provides can be considered as totally novel and can anticipate the behaviour of this antioxidant or other similar ones in other food lipid oxidation processes, and perhaps also in endogenous oxidation. Finally, it only remains to be added that the methodology used in this study covers the monitoring throughout the oxidation process of all the numerous compounds involved in it that can be detected by this technique, regardless of whether they are degraded or formed in it, something which is very difficult if not impossible to achieve with another methodology. In this way, the limitations of previous methods [35,36] are circumvented.

## 2. Materials and Methods

### 2.1. Subject Samples of Study

The subject samples of study are sunflower oil, S, purchased in a local supermarket, and this same oil enriched in hydroxytyrosol acetate (HTy-Ac), in proportions of 0.025% by weight, sample S_025_, of 0.125% by weight, sample S_125_, of 0.250% by weight, sample S_250_, and of 0.750% by weight sample, S_750_. The composition of sunflower oil in fatty acyl groups was estimated by ^1^H NMR, as in previous studies [37,38,39]. Their molar percentages are 57.6 ± 0.3%, of linoleic, 32.0 ± 0.7% of oleic and 10.4 ± 0.5% of saturated acyl groups. The HTy-Ac used to enrich the sunflower oil has a purity of 99.54% and was purchased from Seprox Biotech (Madrid, Spain).

### 2.2. Storage Conditions

Aliquots of 10 g of sunflower oil alone (sample S), and enriched with 0.025%, 0.125%, 0.250%, and with 0.750% by weight of HTy-Ac (samples S_025_, S_125_, S_250_, S_750_ respectively), were poured into glass Petri dishes with 80 mm diameter. These were kept in a convection oven at 70 °C in the presence of air until a very advanced stage of their oxidation process, trying to simulate accelerated storage conditions at intermediate temperature. The experiment was carried out in duplicate. The evolution of each sample under the aforementioned conditions was monitored using ^1^H NMR spectroscopy.

### 2.3. Study of the Samples Evolution by ^1^H NMR Spectroscopy

#### 2.3.1. H NMR Spectroscopy Operating Conditions

The ^1^H NMR spectra of the several samples, S, S_025_, S_125_, S_250_ and S_750_, throughout the storage, were acquired in duplicate by using a Bruker Avance 400 spectrometer operating at 400 MHz. To this aim approximately 175 µL of the abovementioned samples were mixed in a 5 mm diameter tube with 425 µL of deuterated chloroform that contained 0.2% of non-deuterated chloroform and 0.03% of tetramethylsilane (TMS) as internal reference (Cortec, Paris, France). The acquisition parameters were the same as those used in previous studies [40,41]: spectral width 5000 Hz, relaxation delay 3 s, number of scans 64, acquisition time 3.744 s and pulse width 90°, with a total acquisition time of 8 min 55 s. The relaxation delays and acquisition times selected allow the complete relaxation of the protons, the signal areas thus being proportional to the number of protons that generate them, making their use for quantitative purposes possible. The experiments were carried out at 25 °C. The ^1^H NMR spectra were plotted at a fixed value of absolute intensity to be valid for comparative purposes using the MestreNova program (Mestrelab Research, Santiago de Compostela, Spain).

#### 2.3.2. Identification of Components

Identification of components present in the original oil as well as of those generated in the oxidation processes was carried out on the basis of the assignment of the ^1^H NMR signals to the different kinds of hydrogen atoms of the different structures. These signals, their chemical shifts and their assignments to the various hydrogen atoms are given in Tables S1–S14 (Supplementary Material). These assignments were made taking into account previous studies, as indicated in each table, or on the basis of the signals of standard compounds acquired for this study. The latter include: 2*E*-hexenal, 2*E*-heptenal, 2*E*-decenal, 2*E*,4*E*-hexadienal, 2*E*,4*E*-heptadienal, 2*E*,4*E*-decadienal, 4,5-epoxy-2*E*-decenal, 12,13-epoxy-9*Z*-octadecenoic acid methyl ester (isoleukotoxin methyl ester), 2-pentylfuran, 2-ethylfuran, amylformate, and octylformate acquired from Sigma-Aldrich (St. Louis, MO, USA); 9,10-epoxy-12*Z*-octadecenoic acid (leukotoxin), 12,13-*E*-epoxy-9*Z*-octadecenoic acid, 4-hydroxy-2*E*-nonenal, 4-hydroperoxy-2*E*-nonenal, 4-oxo-2*E*-nonenal, 9,10-dihydroxy-12*Z*-octadecenoic acid (leukotoxin diol), 12,13-dihydroxy-9*Z*-octadecenoic acid (isoleukotoxin diol), 9-keto-12,13-*E*-epoxy-10*E*-octadecenoic acid, 9-keto-10*E*,12*E*-octadecadienoic acid, 9-keto-10*E*,12*Z*-octadecadienoic acid, 13-keto-9*Z*,11*E*-octadecadienoic acid and 12R-hydroxy-9*Z*-octadecenoic acid methyl ester (ricinoleic acid methyl ester), purchased from Cayman Chemical (Ann Arbor, MI, USA); 9S-hydroxy-10*E*,12*E*-octadecadienoic acid (Dimorphecolic acid), methyl 13S-hydroxy-9S,10R-epoxy-11*E*-octadecenoate, 11S-hydroxy-9S,10S-epoxy-12*Z*-octadecenoic acid methyl ester, 13S-hydroxy-11S,12S-epoxy-9*Z*-octadecenoic acid methyl ester, 9-hydroxy-10-keto-12*Z*-octadecenoic acid, 9,10-*Z*-12,13-*Z*-diepoxyoctadecanoic acid and 13-hydroxy-12-keto-9*Z*-octadecenoic acid acquired from Larodan (Malmö, Sweeden).

#### 2.3.3. Quantification of the Components

The estimation of the concentration of the different functional groups or groups of compounds present in the several samples, throughout the storage, was possible because the area of each ^1^H NMR spectral signal is proportional to the number of protons that generates it, and this proportionality is constant for all kind of protons. Taking this into account, the estimation of the concentration of the polyunsaturated fatty acyl groups, of HTy-Ac and of the several derivatives in relation to that of triglycerides (TG) was carried out throughout the storage. To this, the area of the signal of the characteristic protons of the several functional groups and that of the protons of the triglycerides, were used. Due to the low level of hydrolysis occurred during oil storage, triglycerides, TG, were taken as an internal reference. Taking into account all of the above, the equation used to estimate the concentrations of the different derivatives was:

[X] = [(A_X_/n)/(A_TG_/4)] 1000, (1)

where “A_X_” is the intensity of the signal selected for the quantification of the X functional group, “n” is the number of protons that generate this signal and “A_TG_” the intensity of the protons at *sn*-1 and *sn*-3 positions in the triglyceride backbone of TG (signal G in Table S1, see Supplementary Material). The area of the signals was determined by using the equipment software and the integrations were performed three times to obtain average values. The concentration thus obtained is expressed in millimole per mol of triglyceride (mmol/mol TG). It is worth noting that, in some cases, the signals of certain compounds overlap with those of others. In these cases, corrections have to be applied in order to avoid underestimations or overestimations of the concentration of the corresponding functional group or derivative of interest.

### 2.4. Statistical Analysis

The data represented in the different figures are average values of at least two determinations and those given in the tables are coming from the aforementioned average values. Microsoft Office Excel 2016 was used to carry out the statistical analysis and for the graphical representation of the obtained values.

## 3. Results and Discussion

As mentioned above, the influence of sunflower oil enrichment in hydroxytyrosol acetate (HTy-Ac) on the evolution of the oxidation of this oil during accelerated storage is the subject of this study. It is well known that when edible oils are subjected to oxidative conditions, they degrade, affecting both their major and minor components, and simultaneously new compounds are formed. In this study, both subjects will be cover successively.

### 3.1. Degradation of Sunflower Oil Main Components and HTy-Ac Added in the Different Samples Submitted to Accelerated Storage

As is known, the main components of edible oils are triglycerides and the main acyl group of sunflower oil is linoleic, as indicated in the Materials and Methods section. Moreover, in sunflower oil, this acyl group is the most unsaturated of all and, therefore, the most easily oxidized. Therefore, the evolution of its concentration during accelerated storage will provide a clear picture of the overall evolution of oil oxidation. As mentioned before, in addition to the sunflower oil (S), four sunflower oil samples enriched with different concentrations of HTy-Ac (S_025_, S_125_, S_250_ and S_750_) were also submitted to accelerated storage conditions and the evolution of the concentration of the linoleic acyl group and of HTy-Ac during storage was studied.

#### 3.1.1. Evolution of the Concentration of Sunflower Oil Linoleic Acyl Group throughout the Accelerated Storage

**Figure 1:**
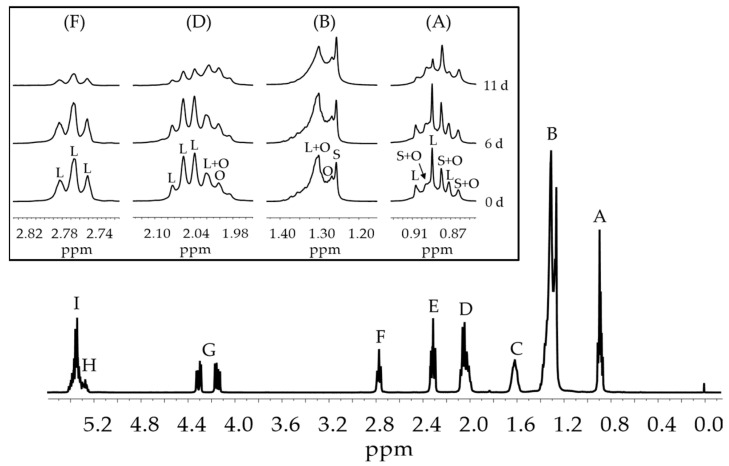
^1^H NMR spectra of sunflower oil S, and the enlargement of signals A, B, D and F, after being submitted to accelerated storage conditions at 70 °C during different periods of time. L: linoleic group; O: oleic group; S: saturated group. The assignment of all signals of this figure is shown in Table S1.

Figure 1 shows the ^1^H NMR spectrum of the unoxidized sunflower oil (S) and some enlarged signals of certain protons of the linoleic acyl group [32,42,43] of the sample S after being subjected during different periods of time to accelerated storage. It can be observed that as the storage time increases the intensity of the signals due to linoleic protons (L) decreases due to its degradation, as is expected.

**Figure 2:**
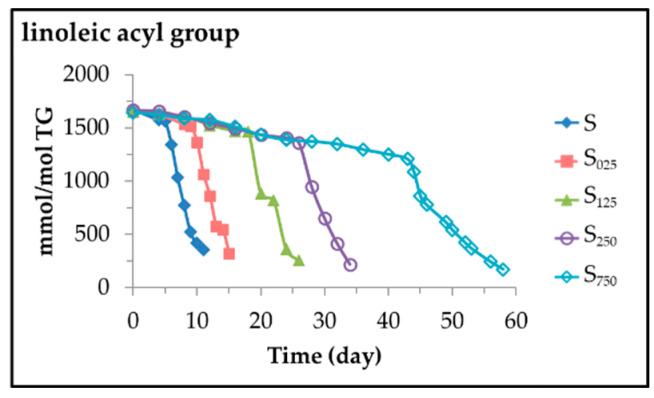
Evolution of the concentration, expressed in mmol/mol TG, of linoleic acyl group in samples S, S_025_, S_125_, S_250_ and S_750_ submitted to accelerated storage at 70 °C up to a very advanced oxidation stage.

From the intensity of signal F of *bis*-allylic protons of linoleic acyl group and that of signal G (see Figure 1) due to some protons of the triglyceride group shown in Table S1, the concentration of linoleic acyl group was estimated in all samples along the storage time. The results obtained for the different samples are depicted in Figure 2.

It can be observed that in all samples, the degradation path of the linoleic acyl group can be considered to be formed by two linear stages, both fit well to a zero order kinetics, with two very different slopes shown in Table 1, which coincide with the degradation rates of the linoleic acyl group (D_R1L_ and D_R2L_) in each stage.

The data represented in Figure 2 and given in Table 1 show that in the oil sample S, the degradation rates of the linoleic group in both stages are much higher than in the other samples. It is evident that the enrichment of sunflower oil in HTy-Ac slows down the rate of degradation of the linoleic group, and therefore of sunflower oil, which leads to a lengthening of the whole oxidation process depending on the level of enrichment, and confirms once again the known antioxidant power of this compound. This slowing down of the rate of linoleic degradation occurs in both stages, being greater in the second stage than in the first. The lengthening of the oxidation process is so great that in the sample most enriched in HTy-Ac, S_750_, the entire oxidation process takes about 58 days compared to 11 days in the S sample.

#### 3.1.2. Evolution of the HTy-Ac Concentration over the Storage Time in the Different Samples

**Figure 3:**
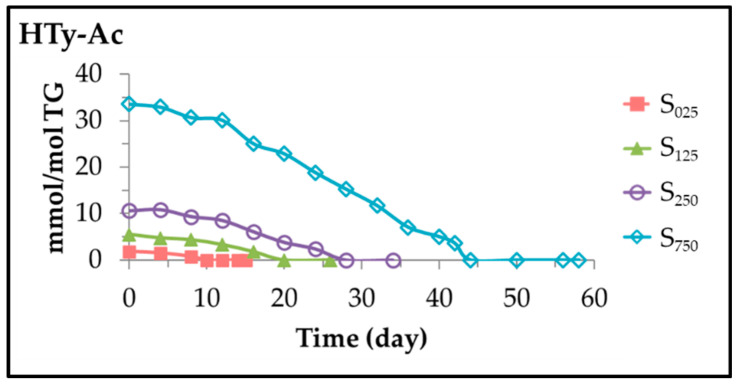
Evolution of the concentration of HTy-Ac, expressed in mmol/mol TG, in samples S_025_, S_125_, S_250_ and S_750_ over the storage time at 70 °C, up to a very advanced oxidation stage.

As mentioned above, under accelerated storage conditions, in the sunflower oil samples enriched in HTy-Ac, this latter compound is also degraded. Its concentration throughout the storage can be estimated, from the intensity of its ^1^H MMR spectral signal centred at 6.77 ppm (see Table S2), and it has been depicted versus time for the different samples in Figure 3.

It can be observed in this figure that the HTy-Ac degradation path can also be considered to be formed by two linear stages, both fit well to a zero order kinetics, with two very different slopes (that are shown in Table 2) which agree with the rates of degradation of HTy-Ac (D_R1H_ and D_R2H_) in each stage. These results are in agreement with those observed in previous studies on natural antioxidants contained in edible oils [31].

Furthermore, data in Table 2 show that both stages are longer the higher the HTy-Ac concentration, with the duration of the first stage being shorter than that of the second stage. Moreover, it is worth noting that in all samples there is the same ratio between the degradation rates of both stages, D_R2H_ being three times greater than D_R1H_.

From observation of Figure 2 and Figure 3, and comparison of data in Table 1 and Table 2, it is clear that the first stage in the degradation pathway of the linoleic acyl group takes the same time as the total degradation of HTy-Ac. In addition, it can also be observed that after total degradation of HTy-Ac, the degradation rate of linoleic acyl group is much higher than when HTy-Ac was present in the sample, that is to say D_R2L_ is higher than D_R1L_ in each sample (see Table 1). Likewise, it is also true, as Table 1 shows, that the degradation rate of the linoleic acyl group in this second stage, D_R2L_, is smaller the greater the original enrichment level in HTy-Ac of the oil sample.

From these results it is evident that the initial concentration of HTy-Ac in the oil sample, as expected, has great influence on the degradation rates of linoleic acyl group in both stages of its degradation pathway, and ultimately on the course of the sunflower oil oxidation. In order to analyze this in detail, possible relationships between the degradation rates of the linoleic acyl group in each one of its degradation stages, D_R1L_ and D_R2L_, and the initial concentration of hydroxytyrosol acetate [HTy-Ac] in the different samples have been studied, and the following equations describe quite well the close relationships between the two sets of parameters.

D_R1L_ = 10.5 − 1.1 Ln [HTy-Ac], R = 0.9932, *n* = 5 (2)

D_R2L_ = 202.7 e^−1.5 [HTy-Ac]^, R = 0.9873, *n* = 5 (3)

**Figure 4:**
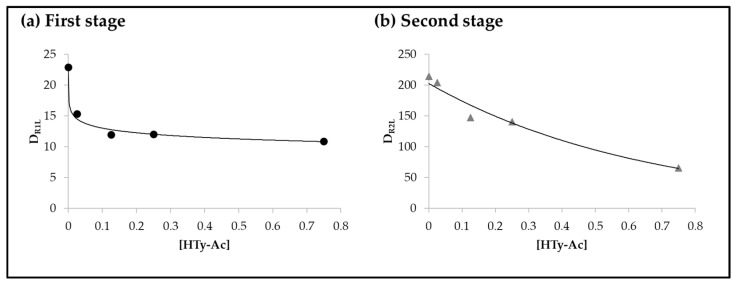
Representation of the degradation rate of linoleic acyl group D_R1L_ in the first (**a**) and D_R2L_ in the second (**b**) stage of its degradation pathway, expressed in mmol mol^−1^ TG day^−1^, versus the initial concentration of the antioxidant [HTy-Ac], expressed in percentage by weight, in the different samples. The lines of these figures correspond to the representation of Equations (2) (**a**) and (3) (**b**) respectively.

These equations are represented by the corresponding lines in Figure 4.

Equation (2) and Figure 4a indicate that the degradation rate of the linoleic acyl group in the first stage of its degradation pathway D_R1L_ is lower the higher the HTy-Ac enrichment of the oil, evidencing the role of this compound as an antioxidant. This equation also shows that the dependence of the first parameter with respect to the second is fairly well described through a Napierian logarithmic function. This indicates that above a certain level of HTy-Ac enrichment, higher levels of enrichment become much less effective in reducing the rate of linoleic acid degradation, or in other words, oil degradation.

Equation (3) and Figure 4b refer to the degradation rate of the linoleic acyl group in the second stage of its degradation pathway, D_R2L_, and its relation with the HTy-Ac enrichment level of the oil sample. It must be remembered that in this second stage of the degradation pathway of the linoleic acyl group, HTy-Ac is not present in the oil sample, due to its previous degradation as mentioned above. This equation, like Equation (2), indicates that the higher the antioxidant enrichment of the oil sample, the lower the rate of linoleic acyl group degradation in this second stage. It should be noted that despite the absence of antioxidant in the oil sample during this stage, the initial level of enrichment of the oil sample in HTy-Ac is highly correlated with the rate of linoleic acyl group degradation at this stage D_R2L_. This relationship is fairly well described by the exponential Equation (3), which indicates at least four important facts. First, that after total degradation of the antioxidant, the oxidation rate of the oil is not the same as that of the unenriched oil sample, nor is it higher, contrary to previously established ideas [33]. Second, that the evolution of the oil oxidation after the total antioxidant degradation is closely associated with the initial enrichment of the oil in antioxidant. Third, that after total destruction of the antioxidant, the rate of degradation of the linoleic acyl group, and therefore of the oil, is lower the higher the level of antioxidant enrichment of the oil. Fourth, that in this second stage, the influence of the initial enrichment level in antioxidant of the oil on the degradation rate of the linoleic acyl group, or, in other words, on the rate of degradation of the oil, is even greater than in the first stage when the antioxidant is present in the sample. This latter is evidenced because D_R1L_ is related inversely with [HTy-Ac] through a Napierian logarithmic function whereas D_R2L_ is related inversely with [HTy-Ac] through an exponential function.

These results demonstrate that, under the conditions of this study, the enrichment of the sunflower oil in HTy-Ac affects not only the initiation phase of its oxidation process, as previously postulated [33], nor only the initiation and propagation phases of this process as recently reported [34], but the entire oxidation process. The technique used and the methodology applied have made it possible to obtain these results, which are very difficult, if not impossible, to achieve using other techniques and methods.

### 3.2. Influence of Sunflower Oil Enrichment in HTy-Ac on Oxylipins Formation and Evolution of Their Concentration throughout the Accelerated Storage

The degradation of the major components of the oil leads to the formation of other compounds, and the nature of these compounds and the evolution of their concentration throughout the oxidation process constitute a source of information of the first order, to know the global oxidation process. However, the study of the oxidation process from this point of view is not easy due to its great complexity, since it involves a host of reactions occurring simultaneously.

As demonstrated above, the sunflower oil enriched in HTy-Ac is oxidized under accelerated storage conditions and, simultaneously with the degradation of the main oil components, oxylipins will be formed. Considering the strong influence that HTy-Ac enrichment has on the degradation of main oil components, it is also expected to affect the formation of oxylipins and the evolution of their concentrations throughout accelerated storage. This is of great interest because of the potential biological effects that some of the oxylipins that may be formed may have.

Due to the complexity of the oil oxidation process and the large number of oxylipins that can be formed and detected by ^1^H NMR, they have been grouped into three groups according to the common features of their potential origin. The first group includes oxylipins derived from the linoleic acyl group that maintain the original chain length, the hydroperoxy conjugated dienes being the parent molecules. The second group includes oxylipins formed by cleavage of other oxylipins and, in some cases, after further evolution or molecular rearrangement. The third group, with origin in the epoxidation of the linoleic acyl group, includes oxylipins that maintain the original chain length and others that, in addition, show branching and even potential cross-linking between chains.

#### 3.2.1. Effect of the Sunflower Oil Enrichment in HTy-Ac on the Formation of Long Chain Oxylipins with Origin in the Peroxidation of the Linoleic Acyl Group, and Evolution of Their Concentration over the Storage Time

**Figure 5:**
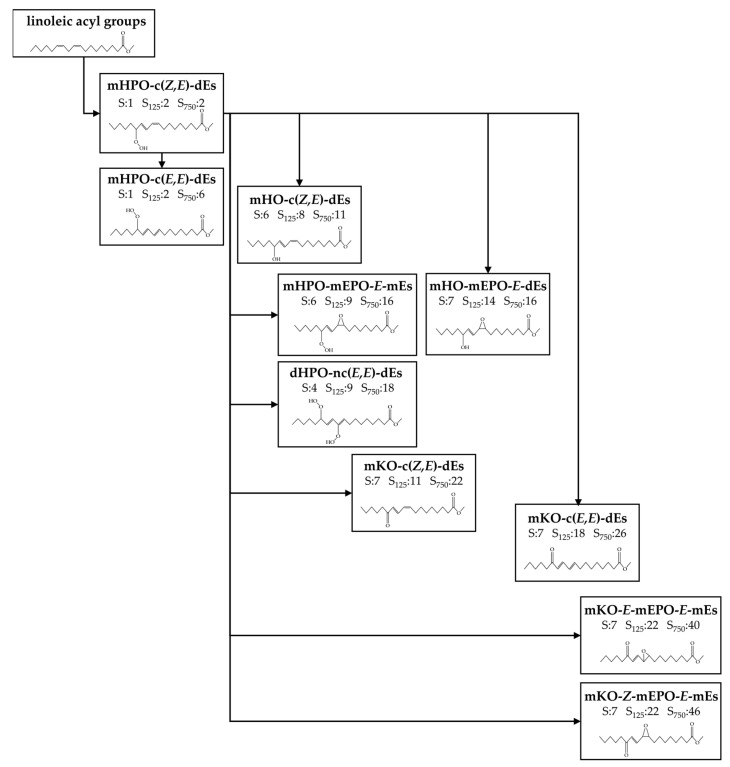
Long-chain oxylipins that have their origin in the peroxidation of the acyl linoleic group, with an indication of their potential precursor according to some authors [44,45,46,47], ordered from left to right and from top to bottom according to the beginning of their formation in samples S_125_ and S_750_ respectively.

Figure 5 shows a scheme of the oxylipins included in this group formed in S, S_025_, S_125_, S_250_ and S_750_ samples over the storage time, with indication of some of their potential precursors, and the day of their formation onset in three of these samples. All details about the influence of the sunflower oil enrichment in HTy-Ac on their formation are explained below.

(A) Monohydroperoxy conjugated dienes (mHPO-c-dEs).

**Figure 6:**
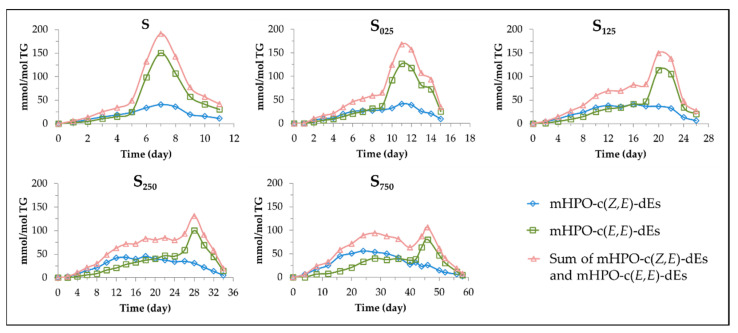
Evolution of the concentration of mHPO-c(*Z,E*)-dEs, mHPO-(*E,E*)-dEs and both types of hydroperoxides together, in samples S, S_025_, S_125_, S_250_ and S_750_ over the storage time at 70 °C, up to a very advanced oxidation stage.

Both mHPO-c(*Z,E*)-dEs and mHPO-c(*E,E*)-dEs are formed in all samples. However, the evolution of their concentration is affected by the level of enrichment of the oil in HTy-Ac. Their concentration during storage was estimated from the intensity of the signals indicated in Table S3 and it is depicted versus storage time for each sample in Figure 6.

As this figure shows, in sample S both types of mHPO-c-dEs are formed in similar concentration for a certain period of time, the concentration of mHPO-c(*Z,E*)-dEs even being somewhat higher than that of mHPO-c(*E,E*)-dEs. Afterwards, the concentration of the latter becomes much higher than that of the former, both simultaneously reaching their maximum concentration, after which both are simultaneously and abruptly degraded. In oil samples enriched in HTy-Ac, the oxidation process is more prolonged the higher the degree of enrichment. Also, the rate of formation of mHPO-c(*Z,E*)-dEs is higher than that of mHPO-c(*E,E*)-dEs during a first period of time, to a greater extent the higher the degree of enrichment in HTy-Ac. Previous studies have shown that this same effect, but to a different extent, is also caused by *gamma* and *alpha* tocopherol in the oxidation of other edible oils subjected to accelerated storage [43,48,49]. It is also worth noting that, at a very advanced oxidation stage, the concentration of mHPO-c(*E,E*)-dEs undergoes a sudden increase in all samples, after which these compounds reach the maximum concentration, followed by a sharp decrease due to their degradation. The onset of the sudden increase in the rate of mHPO-c(*E,E*)-dEs formation occurs in all samples shortly before the total consumption of HTy-Ac and, in the most enriched samples, also coincides with the decrease in the concentration of mHPO-c(*Z,E*)-dEs. Furthermore, the higher the enrichment of the oil in HTy-Ac, the greater the difference in time at which mHPO-c(*Z,E*)-dEs and mHPO-c(*E,E*)-dEs reach their maximum concentration. All these facts suggest that HTy-Ac hinders the isomerization of *Z,E* to *E,E*, to a greater measure the higher the level of HTy-Ac enrichment in the oil. This same effect is also observed, although to a different extent, by *gamma* and *alpha* tocopherol in the oxidation of other edible oils subjected to accelerated storage [43,48,49].

Although in all samples the maximum concentration reached by mHPO-c(*E,E*)-dEs is higher than that reached by mHPO-c(*Z,E*)-dEs, as the degree of enrichment in HTy-Ac increases the concentration of the former decreases considerably whereas that of the latter only increases slightly. These results indicate that HTy-Ac favours an earlier increase in the formation rate of mHPO-c(*Z,E*)-dEs over that of mHPO-c(*E,E*)-dEs, and delays, in general, the degradation of mHPO-c(*Z,E*)-dEs and especially the sudden increase in the formation rate of mHPO-c(*E,E*)-dE until almost the total consumption of HTy-Ac. Furthermore the higher the HTy-Ac enrichment, the lower both the rate of formation of mHPO-c(*E,E*)-dEs and the total concentration of mHPO-c-dEs, and the slower the oxidation process. Likewise, the higher the enrichment in HTy-Ac, the longer the mHPO-c-dEs will be present in the lipid system and the later their transformation into secondary oxidation compounds will take place. These results have shown that HTy-Ac enrichment does not avoid oil oxidation but slows it down and affects the whole oxidation process before and after the total degradation of HTy-Ac, in agreement with above findings. Finally, it only remains to be added that the effects caused by the enrichment of the oil in HTy-Ac on the formation of primary oxidation compounds are also transferred to secondary and further oxidation compounds, some of which are shown in Figure 6, as will be explained below. Several biological activities of these compounds have been the subject of study [50,51,52,53,54] among which can be cited the ability to relax cells in canine arteries [55] and to promote intestinal inflammation [56].

(B) Dihydroperoxy non conjugated dienes (dHPO-nc(*E,E*)-dEs).

**Figure 7:**
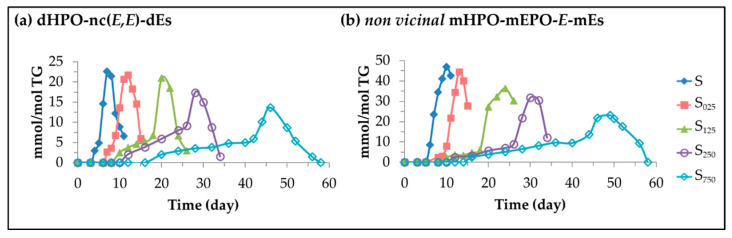
Evolution of the concentration, expressed in mmol/mol TG, in samples S, S_025_, S_125_, S_250_ and S_750_ over the storage time at 70 °C up to a very advanced oxidation stage, of: (**a**) dHPO-nc(*E,E*)-dEs; and (**b**) *non vicinal* mHPO-mEPO-*E*-mEs.

These are secondary oxidation compounds, derived from mHPO-c(*Z,E*)-dEs as has been indicated in Figure 5 [57,58,59,60,61], whose ^1^H NMR signals are given in Table S4. The intensity of their signal near 4.82 ppm has been used to estimate their concentration over the storage time in the different samples, which is depicted versus storage time in Figure 7a. This figure shows that they are formed later and in lower concentrations the higher the enrichment of the oil in HTy-Ac and that their formation starts when the level of degradation of HTy-Ac is not important (see Figure 3 and Table 2). Figure 7a also clearly indicates that dHPO-nc(*E,E*)-dEs are oxidation intermediates as their concentration reaches a maximum after which it decreases sharply due to their degradation, leading to the formation of other oxidation compounds, some of which are well known to be toxic [58,59,60,61]. Their presence in the sample, which coincides over a long period of time with that of their precursors (see Figure 6 and Figure 7a), is more prolonged in time the higher the degree of enrichment in HTy-Ac, reaching their maximum concentration simultaneously with that of mHPO-c(*E,E*)-dEs. This last fact could indicate that the formation of both types of compounds is affected by the same factors. To our knowledge, no biological activities of these oxidation compounds have been described, however, it is important that HTy-Ac enrichment delays and reduces their formation due to the recognized toxicity of some of their derivatives.

(C) *Non vicinal* monohydroperoxy monoepoxy *E*-monoenes (*non vicinal* mHPO-mEPO-*E*-mEs).

The precursors of these compounds are also the mHPO-c(*Z,E*)-dEs as shown in Figure 5 [47,62,63,64,65]. The ^1^H NMR signals of their protons are shown in Table S5 and, from the intensity of their signal centred near 5.85 ppm, their concentration in the different samples over the storage time has been determined, and plotted in Figure 7b versus storage time. It can be observed that their formation also occurs quite early, when HTy-Ac has not yet undergone significant degradation (see Figure 3 and Table 2). There is a very close parallelism between the evolution of the concentration of these compounds throughout storage and that of dHPO-nc(*E,E*)-dEs, as shown in Figure 7a,b, although *non vicinal* mHPO-mEPO-*E*-mEs are formed in higher concentration than the former. Their biological activity has scarcely been studied [52,66], however they are precursors of some well-known toxic aldehydes [63,64,65], so the effect of HTy-Ac enrichment delaying and reducing their formation is very important.

(D) Monohydroxy conjugated *Z,E*-dienes (mHO-c(*Z,E*)-dEs).

**Figure 8:**
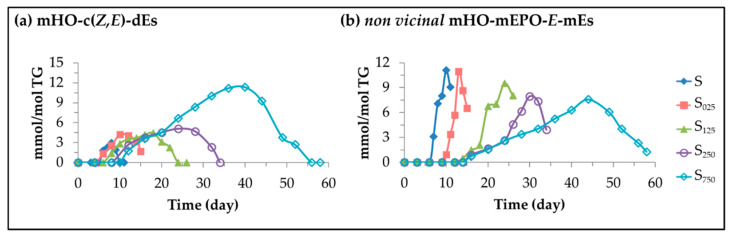
Evolution of the concentration, expressed in mmol/mol TG, in samples S, S_025_, S_125_, S_250_ and S_750_ over the storage time at 70 °C up to a very advanced stage of their oxidation stage of: (**a**) mHO-c(*Z,E*)-dEs; and (**b**) *non vicinal* mHO-mEPO-*E*-mEs.

Figure 8a shows the evolution of the concentration of these compounds in the different samples over the storage time. This has been estimated from the signal intensity of some of their conjugated dienic protons indicated in Table S6. Some of their potential precursors are also the mHPO-c(*Z,E*)-dEs [44,47,67] and, in the samples enriched in HTy-Ac mHO-c(*Z,E*)-dEs are the first secondary oxidation compounds formed (see Figure 5). In accordance with the formation of their potential precursors and contrary to what was observed in the formation of mHPO-c(*E,E*)-dEs, dHPO-nc(*E,E*)-dEs and *non vicinal* mHPO-mEPO-*E*-mEs they reach higher concentrations the higher the enrichment of the oil in HTy-Ac. Their role as intermediate compounds is clear as shown in Figure 8a, with aldehydes being among their derivative compounds, some of them well known to be toxic [68]. As they reach their maximum concentration very close to the total degradation of HTy-Ac, their derived toxic compounds will be formed mainly after this storage time and this occurs later the higher the enrichment in HTy-Ac. In the last time, there has been interest in the biological activity of these compounds [69,70,71,72,73,74,75] and their ability to produce inflammatory hyperalgesia [76], to produce pain [77], as well as to activate trigeminal neurons [78] has been demonstrated.

(E) *Non vicinal* monohydroxy monoepoxy *E*-monoenes (*non vicinal* mHO-mEPO-*E*-mEs).

These compounds are also derived from mHPO-c(*Z,E*)-dEs [44,47] as indicated in Figure 5. Their estimated concentration in the different samples, over the storage time, from the intensity of their spectral signal centred near 5.95 ppm (see Table S7), is depicted in Figure 8b. The formation of these compounds starts very close to that of the *non vicinal* mHPO-mEPO-*E*-mE but with a lower rate of formation, which is why they reach a much lower concentration (see Figure 7b). Nevertheless, the formation paths of both kinds of compounds have in common that the higher the enrichment in HTy-Ac, the later their formation begins, the lower the concentration reached, and both reach their maximum concentration just after the total HTy-Ac degradation, furthermore, as shown in Figure 7b and Figure 8b, both are intermediate compounds. In short, sunflower oil HTy-Ac enrichment delays and reduces the formation of these compounds, which have recently been related with the activation of trigeminal neurons [78] and with the epidermal skin barrier [79].

(F) Monoketo conjugated dienes (mKO-c-dEs).

**Figure 9:**
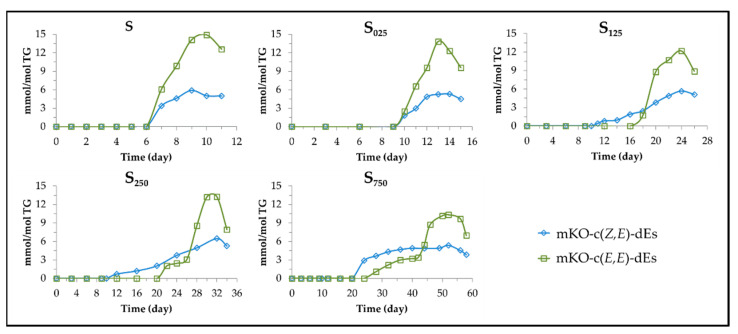
Evolution of the concentration of mKO-c(*Z,E*)-dEs and mKO-(*E,E*)-dEs throughout the storage at 70 °C in samples S, S_025_, S_125_, S_250_ and S_750_ up to a very advanced oxidation stage.

The formation of this type of oxidation compounds, able to be derived from mHPO-c(*Z,E*)-dEs [44,47,67], and from mHO-c(*Z,E*)-dEs [80] occurs, in general, at a more advanced oxidation stage than that of all the aforementioned compounds, as shown in Figure 5. As in mHPO-c-dEs, the two types of isomers mKO-c(*Z,E*)-dEs and mKO-c(*E,E*)-dEs are detected, and as in those, the onset of formation of each isomer and the evolution of its concentration over the storage time is very different in each sample. Their concentration was estimated in all samples from the intensity of the signals indicated in Table S8 and is plotted in Figure 9 versus storage time.

It can be observed in this figure that in samples S and S_025_, the onset of formation of both types of mKO-c-dEs coincides. However, as the oil enrichment in HTy-Ac increases, the formation of mKO-c(*E,E*)-dEs is delayed regarding that of mKO-c(*Z,E*)-dEs. The concentration reached by mKO-c-dEs is low, and that of *Z,E* isomers is lower than that of *E,E* isomers. The concentration of the latter undergoes a sudden increase, coinciding with the almost total degradation of HTy-Ac, in agreement with the occurred in the formation of mHPO-c(*E,E*)-dEs. Both isomers reach their maximum concentration at a very advanced oxidation stage when there is no HTy-Ac left in the system.

Furthermore, and also in agreement with what occurred in the formation of mHPO-c(*E,E*)-dEs, a small reduction in the concentration reached by mKO-c(*E,E*)-dEs is observed as the oil enrichment in HTy-Ac increases. Finally, it only remains to be added that, as Figure 9 shows, these oxylipins are also intermediate compounds, this fact being more evident in mKO-c(*E,E*)-dEs than in mKO-c(*Z,E*)-dEs. From these results, it is clear that the enrichment of sunflower in HTy-Ac has a major influence on the formation of these oxylipins and on all the consequences that follow. Their biological activity has been the subject of interest in recent times [71,81,82,83]. They have been associated with inflammatory hyperalgesia [76] and pain [77], as well as with cytotoxic effects against human ovarian cancer cells [84].

(G) *Non vicinal* monoketo monoepoxy monoenes (*non vicinal* mKO-mEPO-mEs).

**Figure 10:**
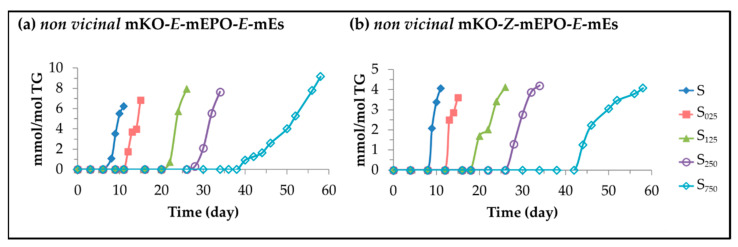
Evolution of the concentration expressed in mmol/mol TG, in samples S, S_025_, S_125_, S_250_ and S_750_ throughout the storage at 70 °C up to a very advanced oxidation stage of: (**a**) *non vicinal* mKO-*E*-mEPO-*E*-mEs; and (**b**) *non vicinal* mKO-*Z*-mEPO-*E*-mEs.

These oxylipins can be derived from mHPO-c(*Z,E*)-dEs [44,45,47], as well as from mHO-c(*Z,E*)-dEs or mKO-c(*Z,E*)-dEs [80]. In the spectra of the different samples, in advanced stages of the oxidation process, characteristic signals of each of the two types of *non vicinal* mKO-mEPO-mEs isomers appear, that is of *non vicinal* mKO-*E*-mEPO-*E*-mEs and *non vicinal* mKO-*Z*-mEPO-*E*-mEs (see Table S9). The concentration of the former was estimated by using the intensity of the spectral signal centred near 3.53 ppm. The concentration of the latter was estimated from the intensity of the signal centred near 3.21 ppm present in the spectra of both types of isomers after subtracting the contribution of the former, assuming that this latter signal is due exclusively to protons from these compounds. The evolution of the concentration so estimated is depicted in Figure 10 versus storage time.

It can be observed in Figure 10 that the onset of the formation of these compounds occurs at a very advanced oxidation stage, coinciding with: the total degradation of HTy-Ac; the onset of the second stage of degradation of the linoleic acyl group; the sudden increase in the concentration of mHPO-c(*E,E*)-dEs, and the decrease in the concentration of mHPO-c(*Z,E*)-dEs. All these facts occur later the higher the HTy-Ac enrichment, these compounds being the last oxylipins formed of all those included in this first group. Both types of mKO-mEPO-*E*-mEs are formed at low concentration, although that of *non vicinal* mKO-*E*-mEPO-*E*-mEs, which increases with the HTy-Ac enrichment, is higher than that of *non vicinal* mKO-*Z*-mEPO-*E*-mEs. Furthermore, from the data depicted in Figure 10 it could be thought that they are end products. It should be highlighted that the influence of oil enrichment in HTy-Ac in delaying the formation of mKO-mEPO-*E*-mEs is very important because several biological activities have been attributed to these compounds. Among them, stimulating the aldosterone production possible related with human hypertension and visceral obesity [85,86,87], provoking itch in skin [88] or activating the trigeminal neurons [78] can be cited.

All of the oxylipins mentioned above could also be formed in other lipid systems that have polyunsaturated omega-6 groups and presumably the effect that HTy-Ac provokes in them could also be as relevant as the one observed in this study.

#### 3.2.2. Effect of the Oil Enrichment in HTy-Ac on the Formation of Oxylipins Coming from the Cleavage of Long Chain Oxidation Derivatives, and Evolution of Their Concentration throughout the Storage Time

In the lipid oxidation processes, under very varied conditions, the formation of small molecules by the cleavage of long chain oxidation compounds, is well known. These cleavage compounds can have very varied functional groups such as acid, alcohol, aldehyde, ketone, furanone, epoxide among others [42,89,90,91], which can be part of small molecules or truncated acyl groups. They can be detected by ^1^H NMR if the signals of their protons do not overlap with those of others and if they are in sufficient concentration to be detected by this technique.

**Figure 11:**
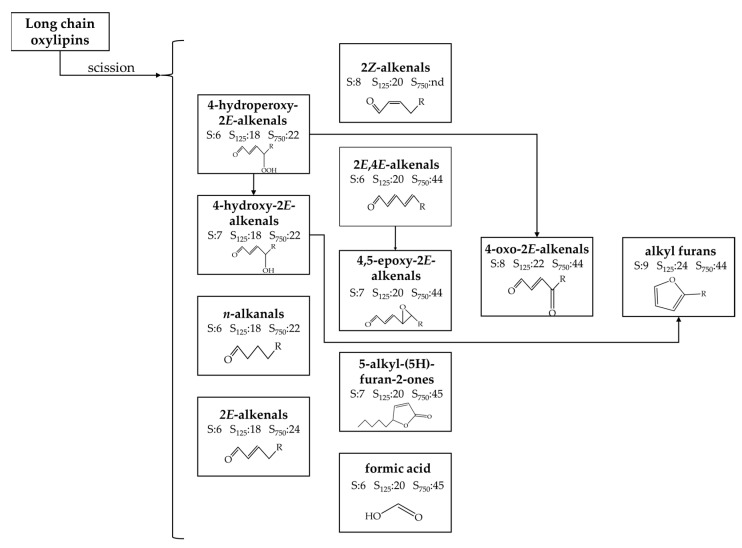
Oxylipins coming from cleavage of long chain oxidation derivatives, with an indication of some of their potential precursors according to certain authors [58,59,68,92,93,94,95,96,97,98], ordered from left to right and from top to bottom according to the beginning of their formation in samples S_125_ and S_750_ respectively after to be submitted to accelerated storage at 70 °C (nd: not detected).

Figure 11 shows the different types of oxylipins coming from long chain cleavage detected with the abovementioned technique in the samples studied here, with an indication, in certain cases, of some of their possible precursors and the first day of their detection under storage conditions in three of the samples subject of study. It can be observed that most of them support the carbonyl group. Among them, three groups have been distinguished, which will be discussed below.

(A) 4-Hydroperoxy-2*E*-alquenals (4HPO-2*E*-alkenals), 4-hydroxy-2*E*-alkenals, (4HO-2*E*-alkenals), *n*-alkanals and 2*E*-alkenals.

**Figure 12:**
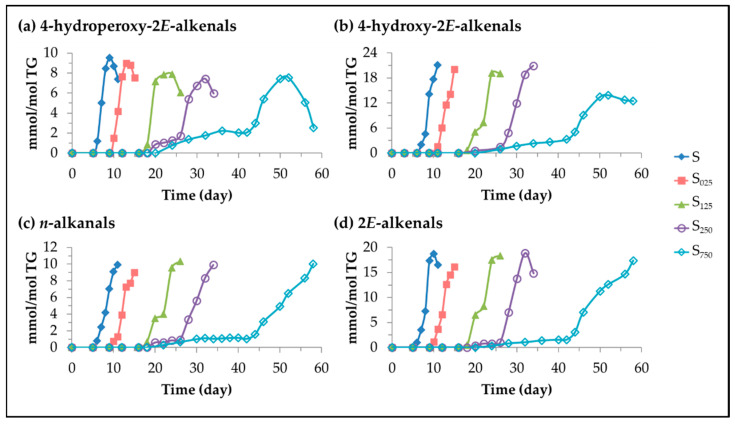
Evolution of the concentration, expressed in mmol/mol TG, in samples S, S_025_, S_125_, S_250_ and S_750_ throughout the storage time, up to a very advanced oxidation stage of: (**a**) 4-HPO-2*E*-alkenals; (**b**) 4HO-2*E*-alkenals; (**c**) *n*-alkanals; and (**d**) 2*E*-alkenals.

Different oxylipins, such as mHPO-c(*Z,E*)-dEs, dHPO-nc(*E,E*)-dEs, mHO-c(*Z,E*)-dEs and *non vicinal* mHPO-mEPO-mEs have been described as precursors [58,59,68,92,93] of these types of aldehydes. It has been reported that the cleavage of these long-chain derivatives results in the formation of two different molecules possessing the aldehyde group. The aldehydic protons of these oxidation compounds give well-known, and non-overlapping ^1^H NMR spectral signals (see Table S10), which allow estimation of their concentration over storage time. Figure 12 shows the evolution of the concentration of these types of aldehydes over the storage time.

In this figure it can be seen that the onset of formation of these four types of aldehydes occurs practically at the same time in each sample, suggesting that they are formed simultaneously, coinciding with the onset of formation of mKO-c(*E,E*)-dEs (see Figure 9). During an initial period of time their rate of formation is low, which is prolonged until HTy-Ac is totally degraded in samples most enriched in this antioxidant. Thereafter there is a steady increase in the concentration of these aldehydes, in all cases reaching the maximum concentration at a very advanced oxidation stage. This figure also shows, especially in the case of the 4-HPO-2*E*-alkenals, that these compounds evolve to give rise to others among which the 4-HO- and 4-KO-2*E*-alkenals have been described [99]. In addition, it is also observed in this figure that the higher the HTy-Ac enrichment of sunflower oil, the later the formation of these compounds occurs, and the lower their formation rate. Of all the aldehydes mentioned above, only 4-HPO-2*E*-alkenals and 4-HO-2*E*-alkenals have relevance from the point of view of biological activity. Both are considered responsible for different degenerative diseases such as atherosclerosis, Alzheimer’s, Parkinson’s and cancer among others, and have received much attention for a long time [89,93,100,101,102,103,104,105,106,107,108,109].

(B) Other aldehydes. 2*E*,4*E*-alkadienals, 4,5-epoxy-2*E*-alkenals (4,5-EPO-2*E*-alkenals), 4-oxo-2*E*-alkenals (4-KO-2*E*-alkenals) and 2*Z*-alkenals.

**Figure 13:**
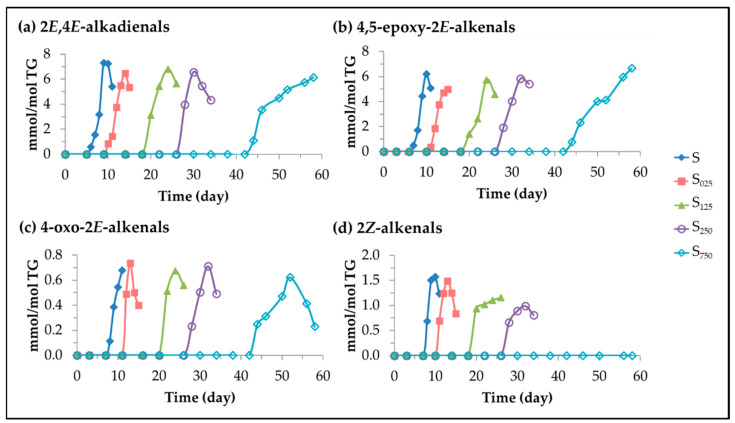
Evolution of the concentration, expressed in mmol/mol TG, in samples S, S_025_, S_125_, S_250_ and S_750_ throughout the storage at 70 °C up to a very advanced oxidation stage, of: (**a**) 2*E*,4*E*-alkadienals; (**b**) 4,5-epoxy-2*E*-alkenals; (**c**) 4-oxo-2*E*-alkenals; and (**d**) 2*Z*-alkenals.

These oxylipins are well known oxidation compounds, and have been detected, in previous studies, by gas chromatography as volatile compounds [42,89,90,91,110,111,112] and by ^1^H NMR [37,113,114,115]. The origin of these oxidation compounds is also in the cleavage of long chain oxidation compounds. Thus, mHPO-c(*Z,E*)-dEs have been described as 2*E*,4*E*-alkadienals precursors and, these latter and also mHPO-mEPO-*E*-mEs have been described as precursors of 4,5-EPO-2*E*-alkenals [94]. Likewise, 4-KO-2*E*-alkenals, as well as 4-HO-2*E*-alkenals, can be derived from 4-HPO-2*E*-alkenals, although the yield of the former is much lower than that of the latter. The concentration of these compounds in the different samples over the storage time was estimated using the intensity of the signals indicated in Table S10, and is plotted versus storage time in Figure 13.

This figure shows that the formation of these aldehydes occurs later than the previous ones and does not start in the samples enriched with HTy-Ac until the total disappearance of this compound. This proves the close relationship between the level of enrichment of sunflower oil in HTy-Ac and the formation of these aldehydes; in fact, the higher the enrichment of the oil in HTy-Ac, the later the formation of these aldehydes starts and the slower the rate of their formation. In the case of 2*Z*-alkenals, the reduction of their formation with HTy-Ac enrichment is so remarkable that they are not detected in the most enriched oil sample. The great reactivity of all these aldehydes and their role as intermediate compounds is also evidenced by the data depicted in Figure 13.

(C) Other oxidation compounds with origin in the cleavage of long chain oxylipins. 5-alkyl-(5H)-furan-2-ones, 5-alkyl-furans and formic acid.

**Figure 14:**
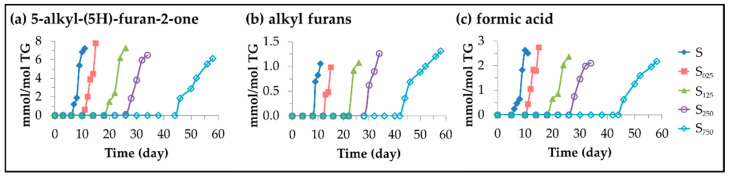
Evolution of the concentration, expressed in mmol/mol TG, in samples S, S_025_, S_125_, S_250_ and S_750_ throughout the storage at 70 °C up to a very advanced oxidation stage, of: (**a**) 5-alkyl-(5H)-furan-2-ones; (**b**) 5-alkyl-furans; (**c**) formic acid.

Well known volatile oxidation compounds [42,91] belong to this group of oxylipins. The first two types are formed after molecular rearrangement of others produced in cleavage processes. The origin of 5-alkylfurans could be in the *alpha*, *beta*-unsaturated aldehydes mentioned above [95], and/or in the 4-HO-2*E*-alkenals after water loss and cyclization [96]. The formic acid formation has been described simultaneously with that of 4-HPO-2*E*-alkenals from mHPO-c(*Z,E*)-dEs [59], and also in the recurrent oxidation of aldehydes [97,98]. The concentration of all of them over the storage time was estimated from the intensity of the signals indicated in Tables S11 and S14 and is plotted versus time in Figure 14.

In this figure one can see that the level of HTy-Ac enrichment of the oil decisively affects the onset of their formation, which occurs after the total degradation of HTy-Ac, as in the last aldehydes discussed. In addition, as the HTy-Ac enrichment increases, a slight decrease in their formation rate is observed.

#### 3.2.3. Effect of the Oil Enrichment in HTy-Ac on the Formation of Oxylipins Derived from Epoxidation of Linoleic Acyl Group Evolution of Their Concentration throughout the Storage Time

**Figure 15:**
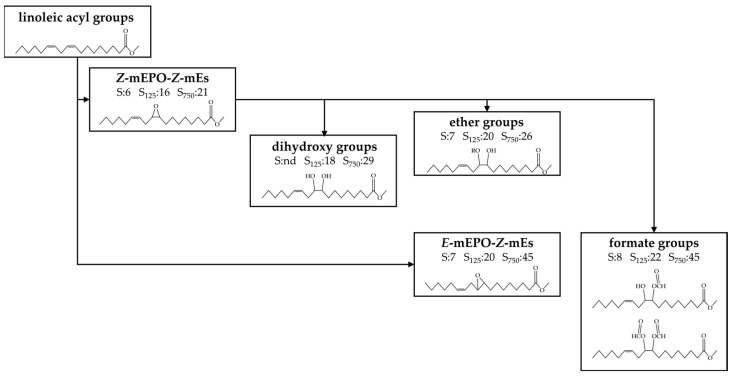
Oxylipins having their origin in the epoxidation of linoleic acyl group and oxirane ring opening, with an indication of the possible precursor [116,117,118,119,120,121,122,123,124,125,126,127,128,129,130,131,132,133] ordered from left to right and from top to bottom according to the beginning of their formation in samples S_125_ and S_750_ respectively, after being submitted to accelerated storage at 70 °C (nd: not detected).

As shown in Figure 2, after the first stage of the linoleic acyl group degradation pathway, which coincides with the storage time at which HTy-Ac is totally degraded, there is still a very important concentration of unmodified linoleic acyl group remaining, which could suggest that other reactions, in addition to those considered above, could take place in the oxidation occurring throughout the storage time. Figure 15 gives a scheme of certain structures, derived from the epoxidation of the linoleic acyl group and subsequent opening of the oxirane ring, with indication of the potential precursor and day of onset of their formation in the different samples. Among them, two groups have been distinguished, which will be discussed below.

(A) Monoepoxy monoenes (mEPO-mEs).

Several mechanisms for the formation of these compounds have been described, among which are the cyclization of alkoxyl radicals [116], or the addition of a peroxyl radical to the double bond of the linoleic acyl group giving rise to the epoxide group and an alkoxyl radical [117,118]. Likewise, the epoxidation on a double bond could also be produced by the concurrence of strong oxidants in the lipid system, such as hydroperoxides, and acids, such as formic acid, which are required factors for the epoxidation reaction, as occurs in the presence of hydrogen peroxide and formic acid [119,120,121].

Regardless of the mechanisms of their formation, the appearance of signals characteristic of long chain oxylipins bearing epoxy groups in the ^1^H NMR spectra of the samples studied, indicate that they are formed throughout the storage. Although the diepoxide formation could occur in the linoleic acyl group, the ^1^H NMR spectra of the samples under study show only very weak signals near 2.99–3.00 ppms attributable to the outer protons of the oxirane rings of diepoxides (see Table S12) at the end of the storage process, which indicates that their formation is very scarce. Due to the low intensity of these signals, they were not quantified because of the unaccuracy of the corresponding results. This indicates that most of the epoxidation reactions occurring in the linoleic acyl group taking place under storage at 70 °C produce monoepoxy monoenes. Spectral signals attributable to epoxy protons from *Z*-mEPO-*Z*-mEs and *E*-mEPO-*Z*-mEs (see Table S12) were detected in the different samples throughout the storage time.

**Figure 16:**
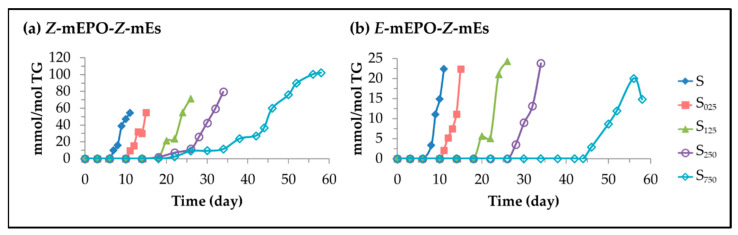
Evolution of the concentration, expressed in mmol/mol TG, in samples S, S_025_, S_125_, S_250_ and S_750_ throughout the storage at 70 °C up to a very advanced oxidation stage of: (**a**) *Z*-mEPO-*Z*-mEs; (**b**) and of *E*-mEPO-*Z*-mEs.

(i) *Z*-Monoepoxy-*Z*-monoenes (*Z*-mEPO-*Z*-mEs). The characteristic signal of the epoxy protons of *Z*-mEPO-*Z*-mEs overlaps with some proton signals of mKO-mEPO-mEs, of 4,5-EPO-2*E*-alkenals (see Tables S12, S9 and S10) and with the side band of the methylene *bis*-allylic protons of the linoleic acyl group (see Table S1). Therefore, to estimate their concentration in the different samples over the storage time, the corresponding corrections were made. The results obtained are depicted in Figure 16a.

The onset of *Z*-mEPO-*Z*-mEs formation (see Figure 16a) coincides with that of *n*-alkanals and 2*E*-alkenals (see Figure 12c,d) and there is also a certain parallelism in the evolution of their concentration since it increases slowly while HTy-Ac is present in the lipid system and increases considerably, in all samples, after total degradation of HTy-Ac.

However, the concentration reached by *Z*-mEPO-*Z*-mEs is much higher than that of these aldehydes, and its increase with enrichment in HTy-Ac is very noticeable, reaching in the most enriched sample, a concentration of a similar order to that of mHPO-c-dEs. The relevance of these findings lies in the fact that *Z*-mEPO-*Z*-mEs includes the so-called leukotoxin and isoleukotoxin [117] that had been considered toxic, responsible for producing some biological effects such as multiple organ failure, and acute respiratory distress syndrome, nowadays only being considered protoxic, these toxic effects being attributed to their corresponding diol derivatives [134].

(ii) *E*-monoepoxy-*Z*-monoenes (*E*-mEPO-*Z*-mEs). As mentioned above, signals attributable to *E*-mEPO-*Z*-mEs are also found in the spectra of the samples under study. This was confirmed using a standard compound and with data provided by other authors (see Table S12) [135]. The concentration of these structures over the storage time in the different samples was determined from the signal intensity indicated in Table S12, and is depicted in Figure 16b. The onset of formation of *E*-mEPO-*Z*-mEs coincides with that of 4,5-epoxy-2*E*-alkenals and 2*E*,4*E*-alkenals (see Figure 11a,b and Figure 16b) and occurs after the total degradation of HTy-Ac. However, the concentration reached by *E*-mEPO-*Z*-mEs is much higher than that of these aldehydes but much lower than that of *Z*-mEPO-*Z*-mEs.

(B) Oxylipins derived from oxirane ring opening.

It is well known that the epoxy group is very reactive and can be opened by different compounds giving rise to *vicinal* dihydroxy groups, or producing branching in the long chains through ester or ether bonds and even potential crosslinking through the formation of ether linkages between long chains.

**Figure 17:**
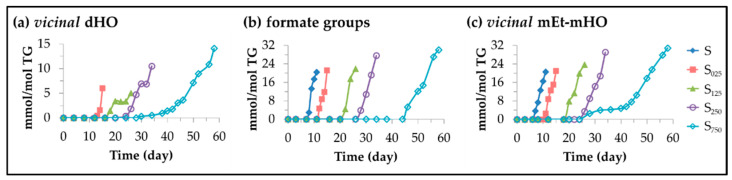
Evolution of the concentration, expressed in mmol/mol TG, in samples S, S_025_, S_125_, S_250_ and S_750_ throughout the storage at 70 °C up to a very advanced oxidation stage of: (**a**) *vicinal* dHO groups; (**b**) formate groups; (**c**) some *vicinal* mEt-mHO groups.

(i) *Vicinal* dihydroxy monoene groups (*vicinal* dHO-mEs). It is common knowledge that the opening of the oxirane ring by hydrolysis produces the corresponding *vicinal* diol groups [122,123]. Their approximate concentration in the samples studied here was estimated from the intensity of the signal centred near 3.43 ppm (see Table S13) after making the corresponding corrections, because protons from other compounds such as *non vicinal* mHPO- and mHO-mEPO-*E*-mEs also contribute to this signal (see Tables S5 and S7) [32], and assuming that any other compound contributes to this signal. The evolution of the concentration so estimated is depicted in Figure 17a.

This figure shows that the formation of *vicinal* dHO groups begins close to the total degradation of HTy-Ac, after the onset of the formation of *Z*-mEPO-*Z*-mEs but before that of *E*-mEPO-*Z*-mEs, which suggests that they derive from the former. The higher the level of enrichment of the sample in HTy-Ac, the later this group forms, reaching a higher concentration, but with a lower rate of formation. It is also remarkable that this group does not form in sample S in accordance with a much lower concentration of its precursors. This is important because these compounds include the known toxicants leukotoxindiol and isoleukotoxindiol mentioned above [117,134,136,137].

(ii) *Vicinal* monoester monohydroxy monoene structures (*vicinal* mEs-mHO-mEs) and/or *vicinal* diester monoene structures (*vicinal* dEs-mEs). Formates (F). The opening of the oxirane ring can also be provoked by acids and, in this case either *vicinal* diester groups or *vicinal* monohydroxy monoester groups can be formed from each opened epoxy group as described in several studies [124,125,126,127,128,129]. The incorporation of acids by ester bonding in the structure of the long chain of the oxidized acyl group gives rise to the branching of this chain. The occurrence of this type of reaction has become evident because the spectra of the samples studied show spectral signals due to the protons of the ester group formed in the case of formic acid, that is to say, of formats. The formate group has characteristic spectral signals (see Table S14), which have been detected previously in studies on polymerization of oils [127], and recently for the first time in ^1^H NMR spectra of edible oils subjected to oxidative conditions [32,115,138]. From their intensity (see Table S14), the formate concentration in the different samples throughout storage was estimated and plotted versus storage time in Figure 17b. This figure shows that the formation of these branchings by formate groups on the long chain of the oxidized acyl groups starts in the samples enriched in HTy-Ac after the total degradation of this compound, the higher the level of the sample enrichment, the greater the delay. It should be noted that the concentration reached by these end products is important, and it should also be remembered that this hydrolysis can also be produced by many other acids which are present in sunflower oil at an advanced stage of oxidation [42]. The effect of branching of long chain oxidized acyl groups is to increase the viscosity of the lipid system as observed in edible oil oxidation experiments.

(iii) *Vicinal* monoether monohydroxy monoene structures (*vicinal* mEt-mHO-mEs). The opening of the oxirane ring by hydrolysis caused by alcohols results in the formation of a *vicinal* monohydroxy monoether group per each open epoxy group [124,126,130,131,132,133]. As already mentioned, in edible oils with a certain degree of oxidation, there are primary and secondary alcohols capable of opening the epoxy group. The opening of the oxirane ring produced by a primary hydroxy group generates branching in the long chain of the oxidized acyl group and when produced by a secondary hydroxy group of another long chain it generates cross-linking between chains. This second possibility gives rise to the oligomerization of the oxidized oil [131]. Evidence that this reaction has taken place is provided by the appearance in the spectrum of a signal centred near 3.61 ppm, attributable to methine protons from secondary alcohols adjacent to an ether group, according to several authors [126,127,139]. Assuming that this signal is due exclusively to this type of protons, the concentration of *vicinal* monoether monohydroxy groups was estimated in all samples and represented versus storage time in Figure 17c.

This figure shows that, as in the case of *vicinal* di-HO groups, the formation of *vicinal* monoether monohydroxy groups begins after the onset of the formation of *Z*-mEPO-*Z*-mEs and before that of *E*-mEPO-*Z*-mEs, suggesting that they derive from the former. The degree of enrichment of the oil in HTy-Ac affects the formation of these compounds in a similar way as it affects the formation of *vicinal* dHO groups; their rate of formation is very small until the total degradation of HTy-Ac, after which it increases continuously until the end of the oxidation process. The consequence of the formation of *vicinal* monohydroxy groups as mentioned above is the branching of the oxidized long chains of the acyl group or crosslinking between them, causing either increased viscosity or polymerization of the lipid system.

### 3.3. Lipolysis Extent and 1,2-Diglycerides Formation throughout the Storage Time

**Figure 18:**
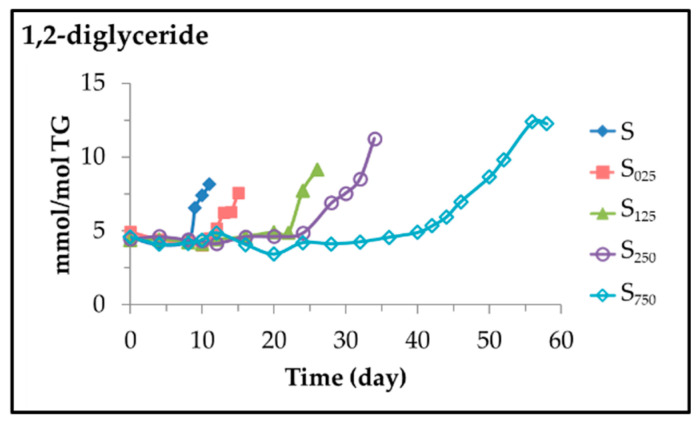
Evolution of the concentration, expressed in mmol/mol TG, of diglycerides in samples S, S_025_, S_125_, S_250_ and S_750_ over the storage time at 70 °C up to a very advanced oxidation stage.

Because of oxidation, in addition to the degradation of both the main components of the oil and the added HTy-Ac, and the formation of a very high number of oxylipins, a lipolytic process also takes place, but of very low extent. This could be expected because in the cascade of reactions that take place in the oxidation process, water is released in some of them. The lipolysis observed refers only to the transformation of triglycerides into 1,2-diglycerides. This has been detected by the increase in the intensity of the signals due to the protons of the latter (see Table S1) in the spectra of the different samples subjected to accelerated storage in relation to the intensity of the same signals present in the original sunflower oil, in all cases referring to that of the triglyceride protons. From the intensity of the signal centred near 3.72 ppm (see Table S1) the concentration of these compounds in the different samples was estimated and is depicted in Figure 18 versus storage time.

This figure shows that sunflower oil, before undergoing oxidation, has a basal concentration of 1,2-diglycerides—close to 5 mmol/mol TG—that remains constant for a long period of time, after which it increases more or less slowly until the end of storage.

The onset of 1,2-diglyceride formation coincides with the almost complete degradation of HTy-Ac and with the onset of the second stage in the linoleic acyl group degradation pathway. To the best of our knowledge, this is the first time it has been demonstrated that both oxidation and lipolysis can occur simultaneously when an edible oil is subjected to accelerated storage conditions. However, it should be added that, as shown in Figure 18, the degree of lipolysis reached is very low and takes place when oxidation is at a very advanced stage.

## 4. Conclusions

As expected, the oxidation rate of sunflower oil subjected to accelerated storage conditions at 70 °C slows down with the enrichment of the oil in hydroxytyrosol acetate. The complete oxidation process takes about five times longer in the oil sample most enriched in this antioxidant than in the non-enriched one. It has been shown that the enrichment of sunflower oil in hydroxytyrosol acetate, under the conditions of this study, does not only affect the first period of the oxidation process also called the induction period as has been pointed out by some authors, nor only the induction and propagation periods of the oxidation process as others have pointed out, but the whole oxidation process. Furthermore, it has also been shown that, after almost complete degradation of the antioxidant, the oxidation rate of the lipid is not equal to or higher than that of the lipid not enriched in antioxidant, but lower, contrary to what has been previously admitted. All of the above has been demonstrated not only globally from the degradation rate of the main component of sunflower oil, the linoleic acyl group, and the added hydroxytyrosol acetate but also from the monitoring of the formation and evolution of the concentration during accelerated storage of all those oxylipins that could be detected by ^1^H NMR spectroscopy.

In all samples the degradation kinetics of the linoleic acyl group fits well to two linear stages, the degradation rates in these two stages being lower the higher the level of enrichment of the oil sample, this effect being much more noticeable in the second stage. Likewise, in all samples, two different stages were found in the degradation kinetics of hydroxytyrosol acetate with the degradation rate of the second stage being about three times the degradation rate of the first stage. Furthermore, the time at which total degradation of hydroxytyrosol acetate occurs coincides with the end of the first stage of the linoleic acid degradation pathway.

As for oxylipin formation, the monohydroperoxide conjugated dienes, mHPO-c-dEs, are present in all samples with varying concentrations throughout the oxidation process. The levels of hydroxytyrosol acetate enrichment tested do not prevent the oxidation of the oil, but slow it down, and this slowing down affects the whole oxidation process, in agreement with what has been observed previously in the degradation of the acyl linoleic group, including the formation of secondary and further oxidation compounds. The maximum concentration reached by mHPO-c(*E,E*)-dEs, in all samples, is higher than that reached by mHPO-c(*Z,E*)-dEs, however as the degree of enrichment in hydroxytyrosol acetate increases, the concentration of the former decreases significantly to nearly half while that of the latter increases only very slightly. In short, the higher the enrichment of the oil in hydroxytyrosol acetate, the lower the formation of total monohydroperoxy conjugated dienes, mHPO-c-dEs, in the oxidation process. Moreover, the presence of the antioxidant favours the early formation of mHPO-c(*Z,E*)-dEs and delays their total degradation, and conversely retards the formation of mHPO-c(*E,E*)-dEs, and especially its sudden increase in concentration that occurs when hydroxytyrosol acetate degradation is almost complete.

The enrichment of the oil samples in the antioxidant favours the formation of monohydroxy-(*Z,E*)-conjugated dienes, mHO-c(*Z,E*)-dEs, although it delays the start of their formation and also their total degradation, this latter almost at the end of the oxidation process in all samples.

The formation and evolution of the concentration of the rest of the oxylipins are also affected by enrichment in hydroxytyrosol acetate and can be grouped into two groups according to the beginning of their formation and the evolution of their concentration. One group includes those oxylipins whose onset of formation occurs well before complete degradation of hydroxytyrosol acetate, except in a reduced number of cases, with a low initial rate of formation that lasts until complete degradation of the antioxidant, after which their rate of formation increases considerably. This group includes dHPO-nc(*E,E*)-dEs, *non vicinal* mHPO-mEPO-*E*-mEs, *non vicinal* mHO-mEPO-*E*-mEs, mKO-c(*Z,E*)-dEs, mKO-(*E,E*)-dEs, *non vicinal* mKO-*Z*-mEPO-*E*-mEs, 4-hydroperoxy-2*E*-alkenals, 4-hydroxy-2*E*-alkenals, *n*-alkanals, 2*E*-alkenals, *Z*-mEPO-*Z*-mEs, *vicinal* dHO, *vicinal* mEt-mHO. Some of these oxylipins are intermediate oxidation compounds. The second group includes those oxylipins whose formation is not detectable until after complete degradation of hydroxytyrosol acetate. This group includes *non vicinal* mKO-*E*-mEPO-*E*-mEs, 2*E*,4*E*-alkadienals, 2*Z*-alkenals, 5-alkyl-(5H)-furan-2-ones, 4,5-epoxy-2*E*-alkenals, formic acid, 4-oxo-2*E*-alkenals, 5-alkyl-furans, *E*-mEPO-*Z*-mEs and formate groups. Furthermore, after complete degradation of the antioxidant, lipolysis of triglycerides to give 1,2-diglycerides also occurs to a very low degree.

To the best of our knowledge, and for the first time, the effect of hydroxytyrosol acetate enrichment of an oil rich in omega-6 groups on the oxidation process that the oil undergoes when it is subjected to accelerated storage has been described, from a double perspective, addressing the evolution of the process as a whole and attending the formation and evolution of the concentration of a considerable number of oxylipins. The effect caused by the presence of hydroxytyrosol acetate, either by preventing the formation of a significant number of oxylipins or by slowing the rate of formation of others, is very important because many of these compounds have been associated with different degenerative diseases.

## Figures and Tables

**Table 1:** Degradation rates (D_R1L_ and D_R2L_), expressed in mmol mol^−1^ TG day^−1^, of linoleic acyl group in the two linear stages of its degradation path depicted in Figure 2, of each of the samples, S, S_025_, S_125_, S_250_ and S_750_, over the storage time at 70 °C. The correlation coefficients of these lines are given in brackets.

Samples	First Stage		Second Stage	
	Time (Days)	D_R1L_ (mmol/mol TG Day)	Time (Days)	D_R2L_ (mmol/mol TG Day)
S	0–5	22.9 (0.98)	5–11	214.0 (0.96)
S025	0–9	15.4 (0.95)	9–15	204.3 (0.98)
S125	0–18	12.0 (0.94)	18–26	147.1 (0.93)
S250	0–26	12.0 (0.98)	26–34	140.8 (0.98)
S750	0–43	10.9 (0.98)	43–58	65.8 (0.94)

**Table 2:** Degradation rates (D_R1H_ and D_R2H_), expressed in mmol mol^−1^ TG day^−1^, of HTy-Ac, in the two linear stages of its degradation path, depicted in Figure 3, in each one of the samples, S_025_, S_125_, S_250_ and S_750_, over the storage time at 70 °C. The correlation coefficients of these lines are given in brackets.

Samples	First Stage		Second Stage	
	Time (Days)	D_R1H_ (mmol/mol TG Day)	Time (Days)	D_R2H_ (mmol/mol TG Day)
S_025_	0–4	0.08 (0.96)	4–10	0.24 (0.96)
S_125_	0–8	0.11 (0.89)	8–20	0.34 (0.98)
S_250_	0–12	0.19 (0.90)	12–28	0.52 (0.99)
S_750_	0–12	0.29 (0.82)	12–44	0.88 (0.99)

## Data Availability

The data presented in this study are available in the article and Supplementary Materials.

## Supplementary Materials

The following supporting information can be downloaded at: https://www.mdpi.com/article/10.3390/antiox11040722/s1, Table S1: ^1^H NMR signals, obtained in CDCl_3_, of protons of main sunflower oil components shown in Figure 1, their chemical shifts, multiplicities and assignments to protons of different functional groups present in edible oils. The signal letters agree with those given in the Figure 1; Table S2: Chemical shift assignments and multiplicities of ^1^H NMR signals in CDCl_3_ of protons of HTy-Ac; Table S3: Chemical shift assignments and multiplicities of ^1^H NMR signals in CDCl_3_ of protons of monohydroperoxy conjugated octadecadienes (mHPO-c-dEs) coming from linoleic groups; Table S4: Chemical shift assignments and multiplicities of ^1^H NMR signals in CDCl_3_ of protons of dihydroperoxy non conjugated *E,E*-octadecadienes (dHPO-nc(*E,E*)-dEs); Table S5: Chemical shift assignments and multiplicities of ^1^H NMR signals in CDCl_3_ of protons of *non vicinal* monohydroperoxy monoepoxy *E*-octadecamonoenes (mHPO-mEPO-*E*-mEs); Table S6: Chemical shift assignments and multiplicities of ^1^H NMR signals in CDCl_3_ of protons of monohydroxy conjugated *Z,E*-octadecadienes (mHO-c(*Z,E*)-dEs); Table S7: Chemical shift assignments and multiplicities of ^1^H NMR signals in CDCl_3_ of protons of monohydroxy monoepoxy *E*-octadecamonoenes (mHO-mEPO-*E*-mEs); Table S8: Chemical shift assignments and multiplicities of ^1^H NMR signals in CDCl_3_ of protons of monoketo conjugated octadecadienes (mKO-c-dEs); Table S9: Chemical shift assignments and multiplicities of ^1^H NMR signals in CDCl_3_ of protons of monoketo monoepoxy *E*-octadecamonoenes (mKO-mEPO-*E*-mEs); Table S10: Chemical shift assignments and multiplicities of ^1^H NMR signals in CDCl_3_ of protons of different types of aldehydes; Table S11: Chemical shift assignments and multiplicities of ^1^H NMR signals in CDCl_3_ of protons of furan groups; Table S12: Chemical shift assignments and multiplicities of ^1^H NMR signals in CDCl_3_ of protons of epoxy derivatives coming from linoleic acyl groups; Table S13: Chemical shift assignments and multiplicities of ^1^H NMR signals in CDCl_3_ of protons of different types of dihydroxy groups (dHO); Table S14: Chemical shift assignments and multiplicities of ^1^H NMR signals in CDCl_3_ of protons of formic acid and formates.
